# Treatment of radiation-induced brachial plexopathy with omentoplasty

**DOI:** 10.4322/acr.2020.202

**Published:** 2020-09-02

**Authors:** Adilson José Manuel de Oliveira, João Paulo de Souza Castro, Luciano Henrique Foroni, Mário Gilberto Siqueira, Roberto Sérgio Martins

**Affiliations:** 1 Universidade de São Paulo (USP), Faculdade de Medicina, Hospital das Clínicas, Instituto de Psiquiatria, Peripheral Nerves Group, São Paulo, SP, Brasil; 2 Clínica Girassol, Neurosurgery Department, Luanda, Angola

**Keywords:** Brachial Plexus Neuropathies, Radiation Injuries, Pain, Intractable, Neurosurgery

## Abstract

Radiation-induced brachial plexus neuropathy (RIBPN) is a rare and delayed non-traumatic injury to the brachial plexus, which occurs following radiation therapy to the chest wall, neck, and/or axilla in previously treated patients with cancer. The incidence of RIBPN is more common in patients treated for carcinoma of the breast and Hodgkin lymphoma. With the improvement in radiation techniques, the incidence of injury to the brachial plexus following radiotherapy has dramatically reduced. The currently reported incidence is 1.2% in women irradiated for breast cancer. The progression of symptoms is gradual in about two-thirds of cases; the patients may initially present with paresthesia followed by pain, and later progress to motor weakness in the affected limb. We present the case of a 68-year-old female patient with breast cancer submitted to surgery, chemotherapy, and radiotherapy in the year 2000. Eighteen years later, she developed symptoms and signs compatible with RIBPN and was successfully submitted to omentoplasty for pain control. Omentoplasty is an alternative treatment for RIBPN refractory to conservative treatment, which seems to be effective in improving neuropathic pain. However, postoperative worsening of the motor strength is a real possibility, and all candidates for this type of surgery must be informed about the risk of this complication.

## INTRODUCTION

Radiation-induced brachial plexus neuropathy (RIBPN) is a delayed non-traumatic injury to the brachial plexus. It is a rare condition that occurs following radiation therapy to the chest wall, neck, and/or axilla in patients previously treated for cancer.^1^ It is defined as the neurologic impairment of a transient or permanent nature involving the brachial plexus as a sequel to radiation treatment. In 1966, Stoll and Andrews^2^ published the first description of this lesion.

The occurrence of RIBPN is higher in patients treated for carcinoma of the breast and for Hodgkin lymphoma. With the improvement in radiation techniques, the incidence of injury to the brachial plexus following radiotherapy has dramatically reduced, and the current incidence in women irradiated for breast cancer is 1.2%.^3^ ^,^ ^4^

RIBPN symptoms can occur from 6 months to 20 years after radiotherapy (usually from 1 to 4 years). The most frequent symptoms are numbness, paresthesia, dysesthesia, lymphedema, and motor weakness. Neurogenic pain is present almost in all cases—the intensity of which varies from mild to incapacitating.^5^ ^,^ ^6^

The progression of symptoms is gradual in about two-thirds of cases. Patients may initially present with paresthesia followed by pain, and later develop motor weakness in the affected upper limb. The pain often subsides when the motor weakness becomes more severe.^7^

For the quantification of normal neurologic tissue damage after radiotherapy, there are several scores. One of the most often used is the LENT–SOMA score: late effects of normal tissues (LENT)–subjective, objective, management, and analytic (SOMA). This score is useful for the stratification and clinical/ surgical management of these patients.^8^

For RIBPN, the LENT–SOMA score has 4 grades and respective approaches. Grade 1: mild sensory deficits—no treatment required; grade 2: moderate sensory deficits, tolerable pain, mild arm weakness—conservative management indicated; grade 3: continuous paresthesia with incomplete paresis—surgical or conservative management may be opted; grade 4: complete paresis, excruciating pain—surgical management required.

### Case Report

A 68-year-old female was diagnosed with breast cancer in the year 2000 and was submitted to a right side quadrantectomy and axillary emptying, chemotherapy, and radiotherapy. The disease was controlled, and no recurrence or intercurrence occurred from 2000 until 2018.

In 2018 the patient started to complain of tingling in the right hand, progressive hypoesthesia in the right upper limb. Six months later, progressive loss of strength in the right upper limb began, which was associated with neuropathic pain in the upper limb (mainly in hand), and lymphedema.

The symptoms became progressively worse and were refractory to the prescribed medications (pregabalin, amitriptyline, dipyrone, and venlafaxine) and physiotherapy.

In 2019 the patient was referred to the Peripheral Nerve Surgery Unit of the Neurosurgery of the University of São Paulo Medical School, with a neuropathic pain visual analog scale (VAS) of 10/10 and exuberant lymphedema in all upper limb. She complained of tingling in the lateral palmar region, the dorsal region of the hand, and the lateral areas of the upper arm and forearm.

On neurologic examination, the motor strength was graded 4 for arm abduction; 3 for arm adduction, elbow extension, and intrinsic muscles of the hand; and 0 for elbow flexion.

With these symptoms and neurological examination, the patient was classified as LENT–SOMA grade 4.

This clinical picture raised the diagnostic hypothesis of RIBPN, and surgical treatment was proposed. The expectation of results and the risk of complications were extensively discussed with the patient.

**Figure 1:**
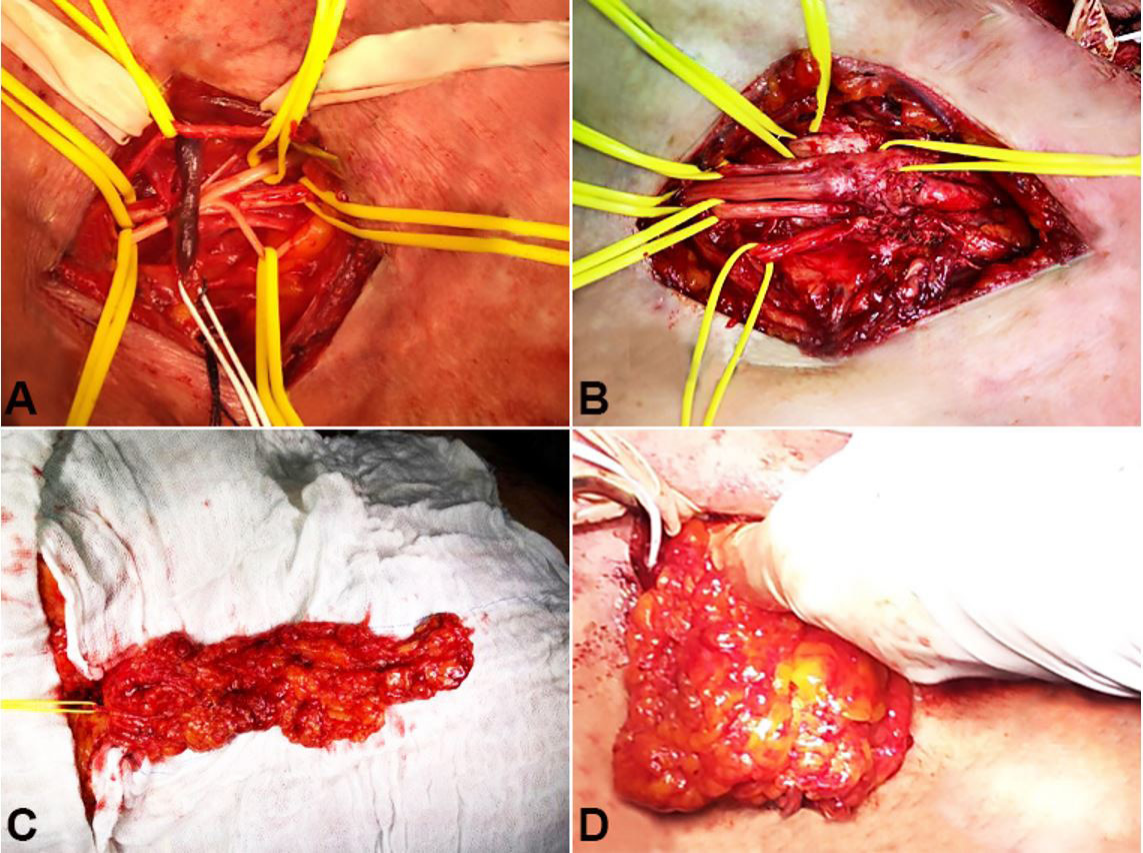
**A –** Intraoperative view of the supraclavicular brachial plexus exploration with surrounding fibrotic tissue, (yellow vessel loop) thrombosed transverse cervical vessel (white); **B -** neuroma in continuity in the lateral cord; **C –** Graft of omentum with vascular pedicle (yellow vessel loop); **D –** covering the brachial plexus with the omental graft.

The patient was submitted to a supraclavicular and infraclavicular surgical exploration of the brachial plexus. The main intra-operative findings were (i) fibrotic tissue adhered to all elements of the supra clavicular brachial plexus (Figure 1A), and (ii) there was a neuroma in continuity in the lateral cord (Figure 1B). Under magnification, an extensive external micro neurolysis was performed. Next, a graft of omentum was harvested through a median supra umbilical laparotomy (Figure 1C), and its vascular pedicle was anastomosed to the cervical transverse vessels. The graft was placed over all elements of the brachial plexus (Figure 1D).

Post-operatively, the patient was submitted to lymphatic drainage and physiotherapy and was discharged on the third postoperative day. At the 6-month follow-up, she was still experiencing tingling in the lateral palmar region, the dorsal region of the hand, and the lateral areas of the upper arm and forearm. The lymphedema improved, and the neuropathic pain achieved (VAS) 0/10, motivating a progressive pain killers’ withdrawal. The patient’s motor strength was equivalent to her preoperative strength in arm adduction (grade 3) and elbow flexion (grade 0); however, it had become worse (grade 2) in arm abduction, elbow extension, and the intrinsic muscles of the hand. With these symptoms and neurological examination, the patient was classified as LENT–SOMA grade 2.

Owing to the disabling effect of the previous neuropathic pain, the surgical treatment was deemed satisfactory, despite the worsening of the motor strength.

## DISCUSSION

The peripheral nerves are considered somewhat radioresistant due to their protected position, low metabolism, and low reproductive capabilities.^9^ ^,^ ^10^ Radiotherapy, usually necessary to treat oncologic patients may present complications caused by the direct effect of radiation in nervous tissue or by the compression caused by the fibrosis surrounding the nervous plexus. In this setting, radiotherapy was reported to increase the risk of brachial plexus lesions.^11^ In our case, both mechanisms - direct neural lesion and adjacent fibrosis played a role in our patient’s neural injury. Also, the patient was previously submitted to chemotherapy.

Chemotherapy was also reported to increase the incidence of RBP significantly. It is believed that, in this setting, the lesion happens by a direct mechanism of chemo in the vessels, and due to a reduction in the metabolism of a tissue that has an inherent low metabolic rate.^3^

RIBPN is a rare but devastating complication that can disable the patient.^9^ There is no consensus regarding the definitive treatment of RIBPN. The options are mainly directed toward controlling the presenting symptoms, such as pain, paresthesia, and psychological disorders.^12^

In our case, the patient presented a typical clinical course of RIBPN with neuropathic pain as the most disabling symptom. The total or partial pain control was the focus of the treatment.

Surgical exploration is fully justified in grades 3 and 4 of the LENT–SOMA score.^10^

The rationale for surgical exploration in patients with RIBPN is the mechanism of lesion – direct lesion to (i) the nerve, (ii) surrounding soft tissues with fibrosis and (iii) vascular lesion at the vasa nervorum. These three injury components promote the neuropathic pain due to compression of brachial plexus, sensory and motor deficits by neuropathy, which is worsened by hypoxia – vascular lesion.^3^ ^,^ ^6^ ^,^ ^7^ ^,^ ^9^

In an attempt to manage the physiopathological mechanisms, the surgical exploration of the plexus, neurolysis with a free graft (muscle or omentum), or pedicled omentoplasty has shown positive results.

In 1983, Narakas^13^ proposed the omentoplasty to treat RIBPN in a series of 45 cases. In this study, the omentoplasty was performed in 15 cases, which has a better result than the other 30 cases that were singly treated with neurolysis. In the Narakas study, the chief result was pain relief.

After Narakas experience, a few case reports or small case series were performed. However, we based our therapeutic option for our patient in the Brunelli and Brunelli^14^ study that comprised 67 cases. In this study, 39 were surgically treated, 3 cases neurolysis, 2 neurolysis, and skin flap, 3 neurolysis metastasis-closure, and 31 omentoplasty.^14^ The criteria for surgical indication in this series was similar to our case (neuropathic pain). Regarding the neuropathic pain, the Brunelli’s results showed partial relief of pain in 28 cases and total relief in the remaining 3 cases.

In 2019, Warade et al.^12^ published a series comprising 11 cases of females with RIBPN (neurogenic pain - visual analog scale 9-10) previously treated for breast carcinoma. In this series, all patients were treated surgically, and neurolysis was undertaken. 81% of the patients presented a significant improvement of neurogenic pain and paresthesia.

We believe that omentoplasty is the best choice for surgical treatment of RIBPN based on Brunelli and Brunelli^14^ experience and in the physiological mechanism of this procedure – the flap of omentum with pedicle improves the mobilization of the brachial plexus without lesion accompanied by the neurolysis, the vessels of the omentum improves circulation e hypoxia.^3^

Despite the absence of strong evidence, we support our decision in some previous case series that have suggested that it is the best alternative for refractory pain relief, although the results for motor strength recovery are poor.^14^ ^,^ ^15^

There is a paucity of published reports related to omentoplasty; we believe that the publication of this case can help doctors dealing with this type of problem. This case could assure them (and their patients) that despite the fact that in most cases there is no improvement in—and even some worsening of—the motor deficit (as in our case), post-operatively the effect on the neuropathic pain is usually good, with a decrease in its intensity or even with complete control.

The expected surgical outcome was widely discussed with the patient, and all ethical aspects were respected for the case publication.

## CONCLUSION

Omentoplasty is an alternative treatment of RIBPN refractory to conservative treatment. It is a safe and effective treatment to improve neuropathic pain; however, worsening of motor strength is a real possibility, and the patient must be alerted to this before the surgery occurs.
